# Supplementary figures and images for: Activated Gab1 drives hepatocyte proliferation and anti-apoptosis in liver fibrosis via potential involvement of the HGF/c-Met signaling axis
Source: PLoS One. 2024 Jun 27;19(6):e0306345. doi: 10.1371/journal.pone.0306345 (PMC11210754; doi:10.1371/journal.pone.0306345)

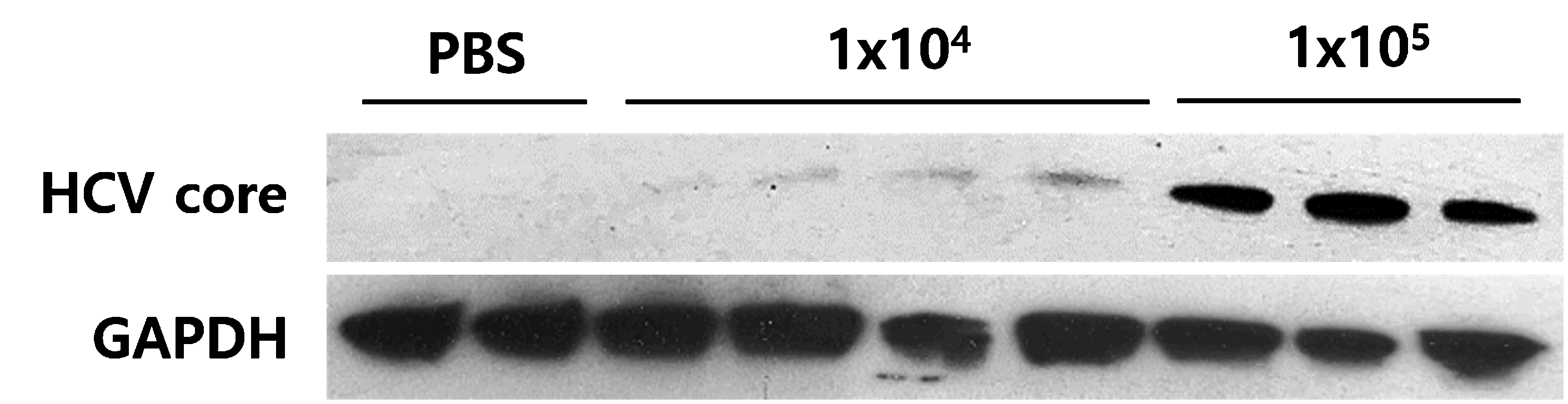

Supplement: S1 Fig — Humanized liver mice were injected in the tail vein with 1 × 10^4^ and 1 × 10^5^ FFU/100 μL of JFH-1 (genotype 2a) diluted in PBS (3 mice/group). Liver tissues were harvested 7 days after virus inoculation. Protein levels of HCV core and GAPDH were analyzed by Western blot. (TIF) [file pone.0306345.s001.tif]
