# Supplementary material for: Activated Gab1 drives hepatocyte proliferation and anti-apoptosis in liver fibrosis via potential involvement of the HGF/c-Met signaling axis
Source: PLoS One. 2024 Jun 27;19(6):e0306345. doi: 10.1371/journal.pone.0306345 (PMC11210754; doi:10.1371/journal.pone.0306345)

Fig 3E

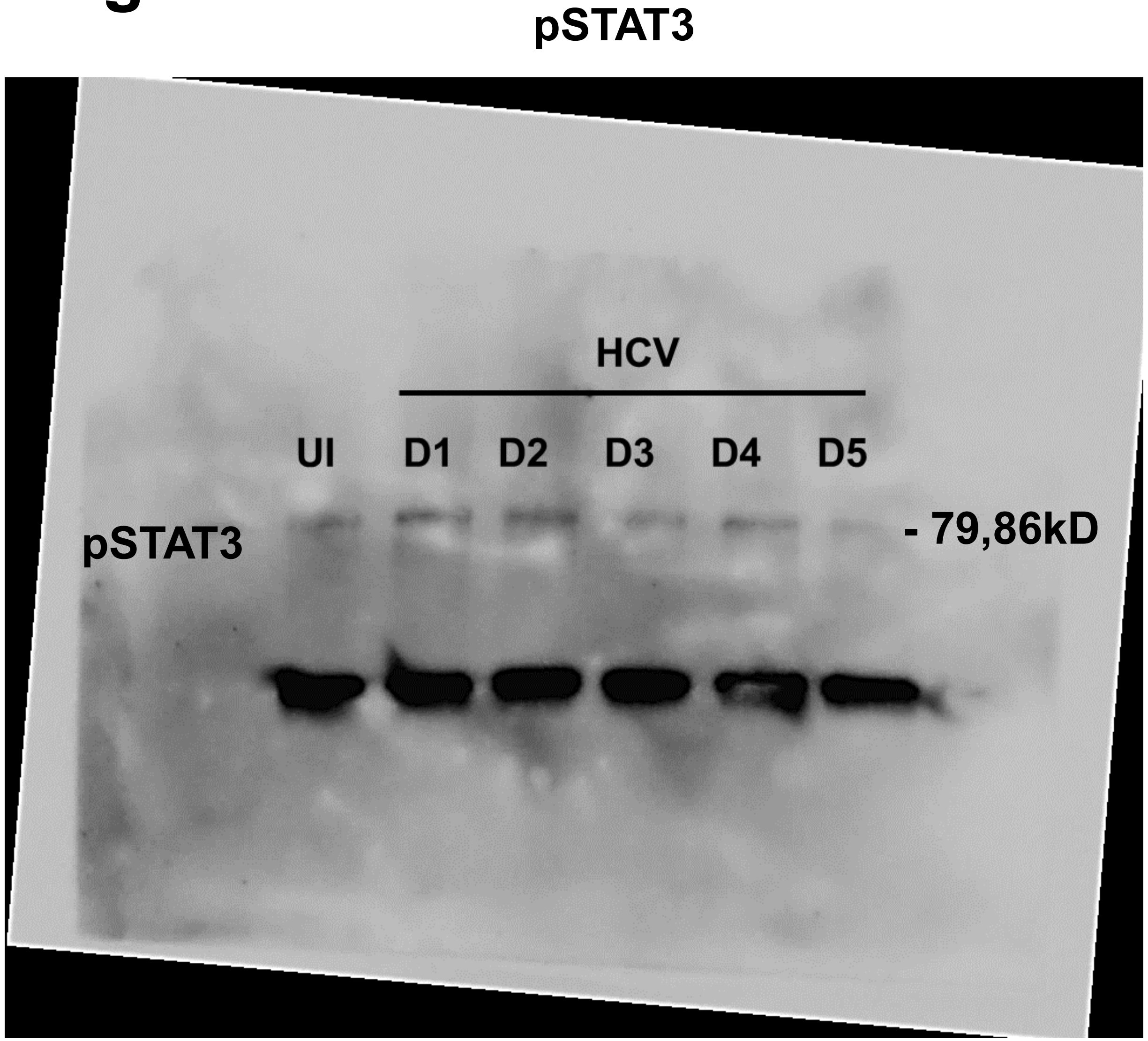
pSTAT3
HCV
UI
D1
D2
D3
D4
D5
pSTAT3
- 79,86kD

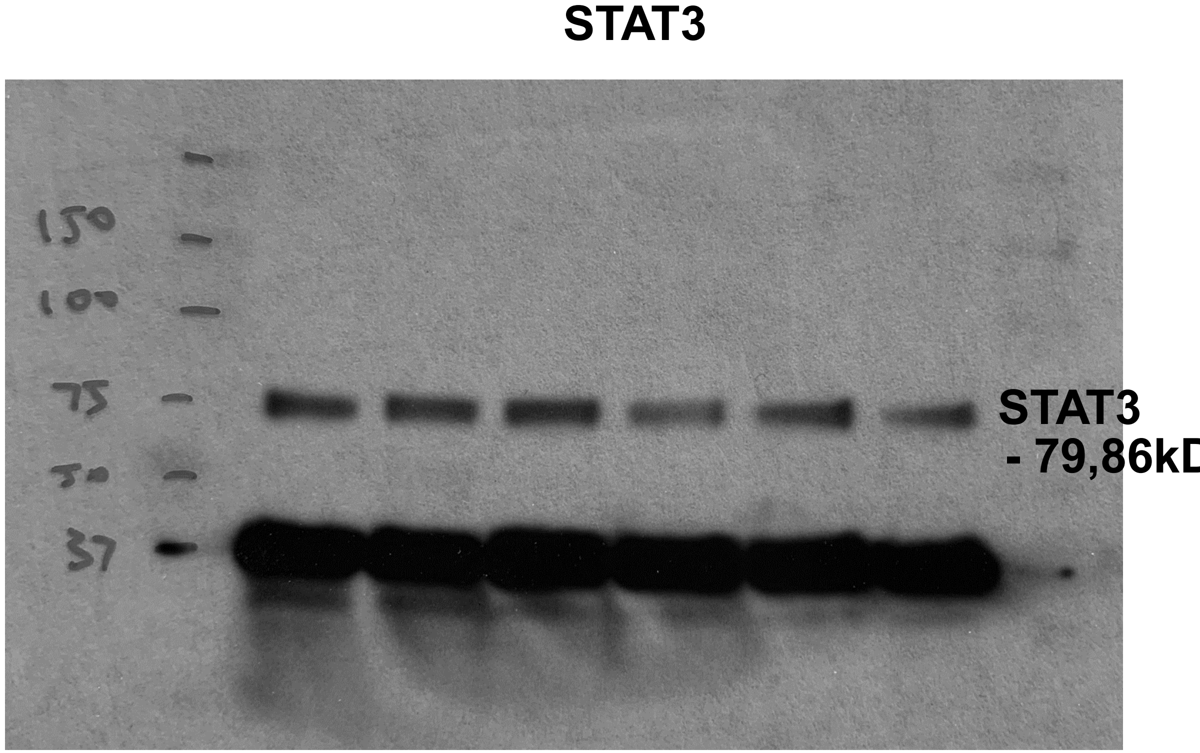
STAT3
150
100
75
50
37
STAT3
- 79,86kD

Fig 3E

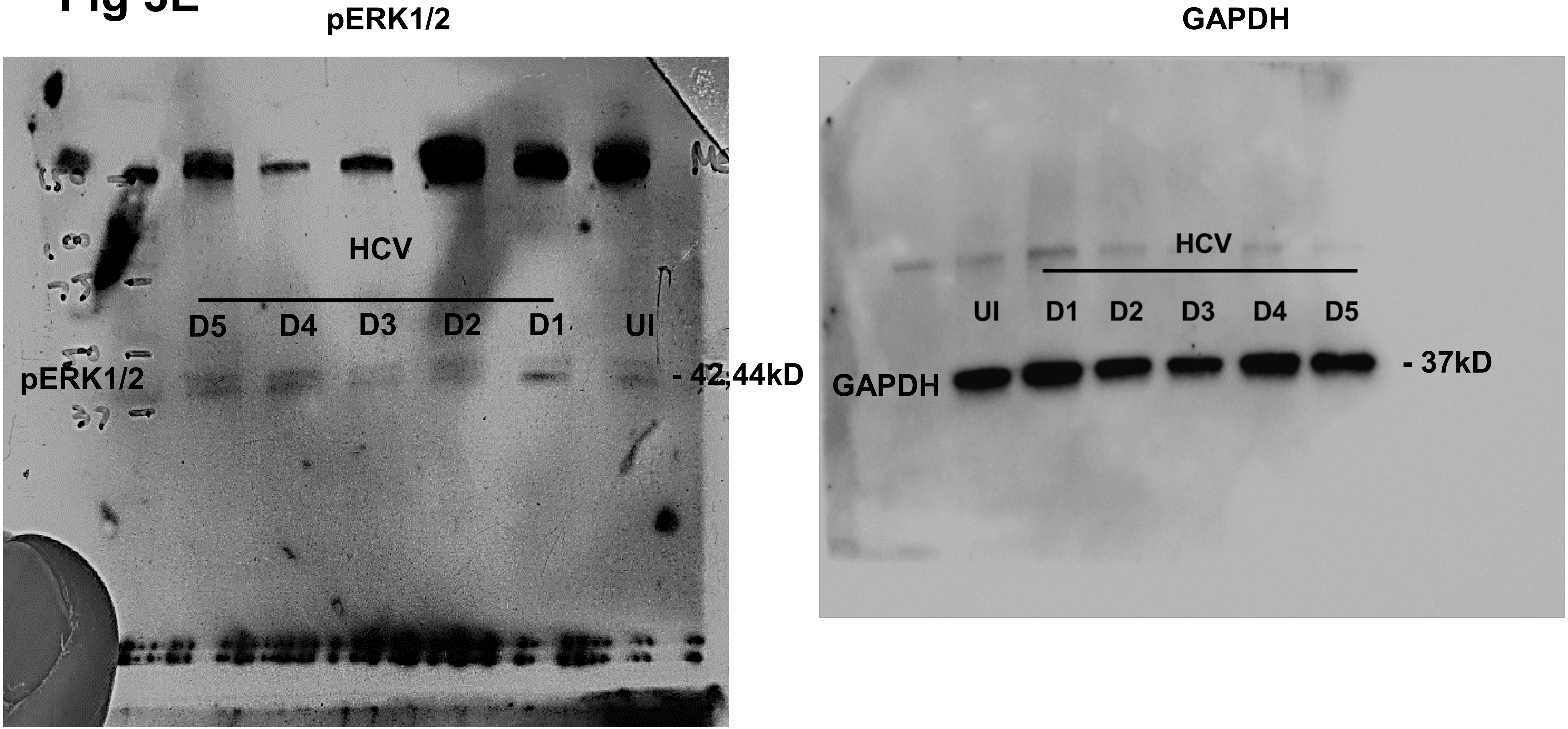
pERK1/2
HCV
D5
D4
D3
D2
D1
UI
pERK1/2
- 42,44kD
GAPDH
HCV
UI
D1
D2
D3
D4
D5
GAPDH
- 37kD

Fig 4D

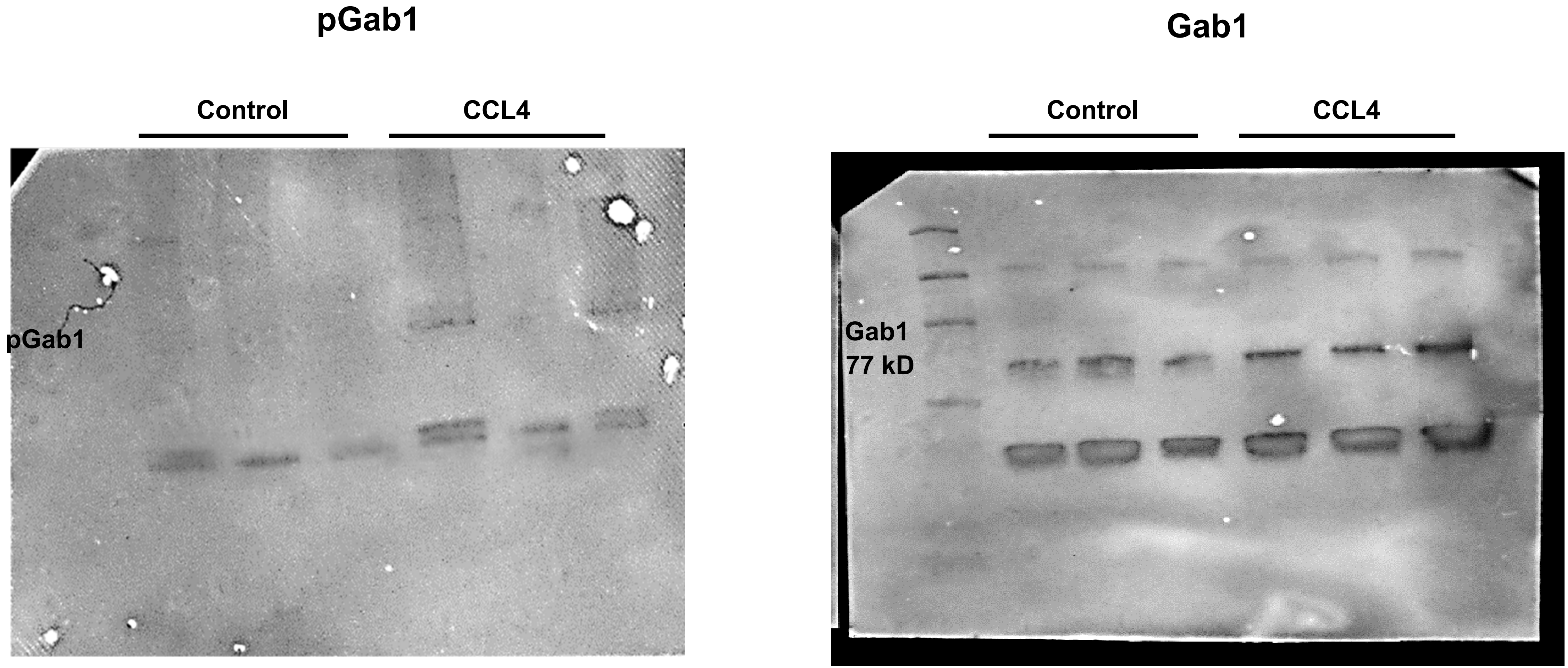

Fig 4D

Gapdh

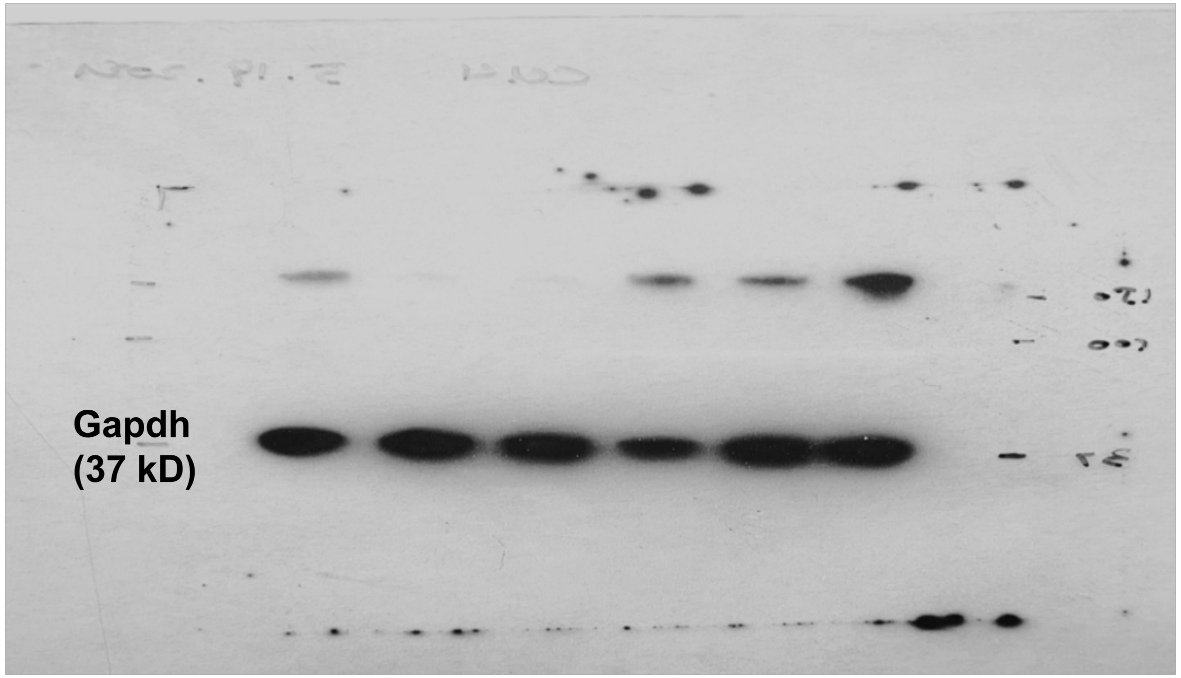

Fig 4F

Ki67

Ki67
(360kD)

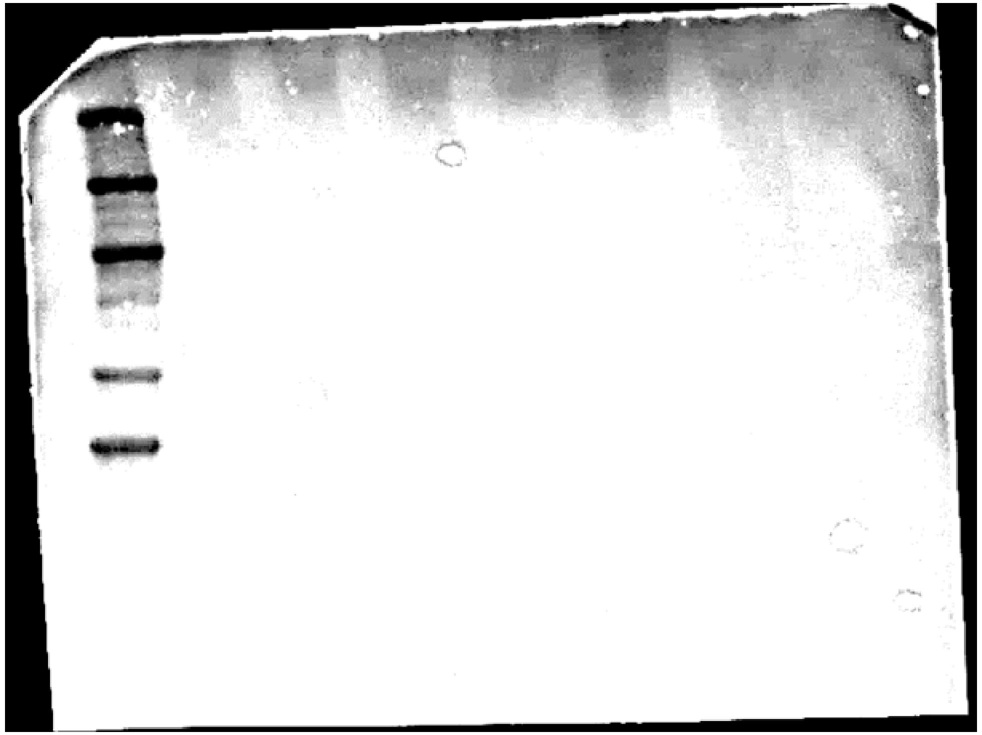

pSTAT3

pSTAT3
(79,86 kD)

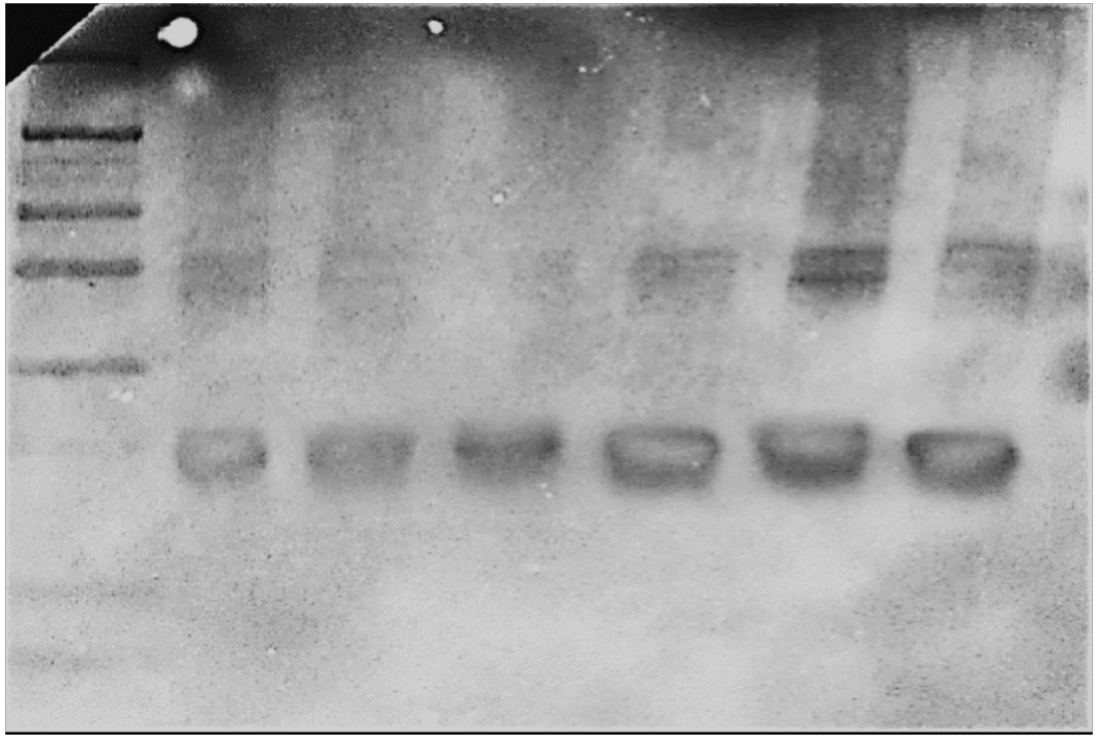

Fig 4F

CYP2E1

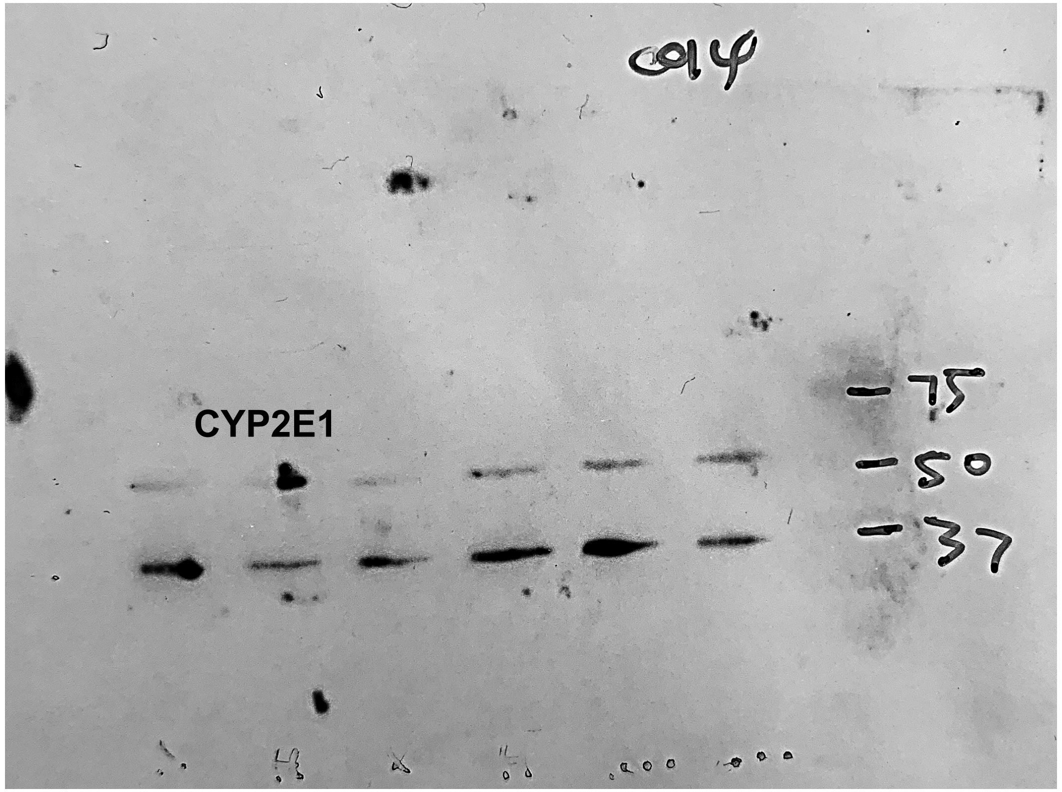

pERK1/2

pERK1/2: These are pERK1/2 bands from the first WB. Since pERK1/2 signal was weak, WB was repeated. The pERK1/2 band from repeated WB was presented in Fig 4D. However, we failed to locate data of repeated WB.

Control CCL4

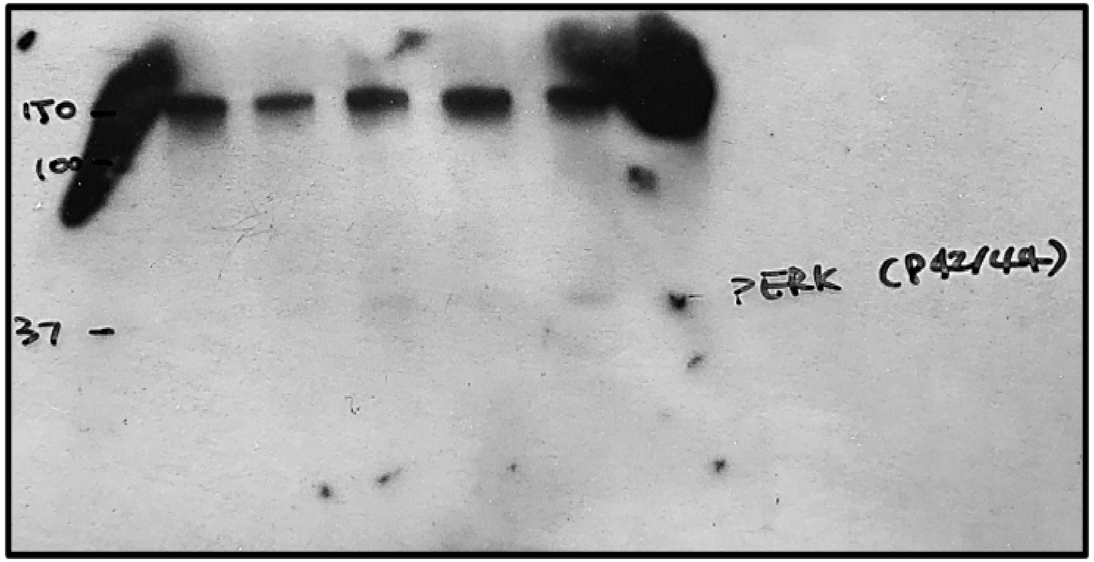

Fig 4F

GAPDH

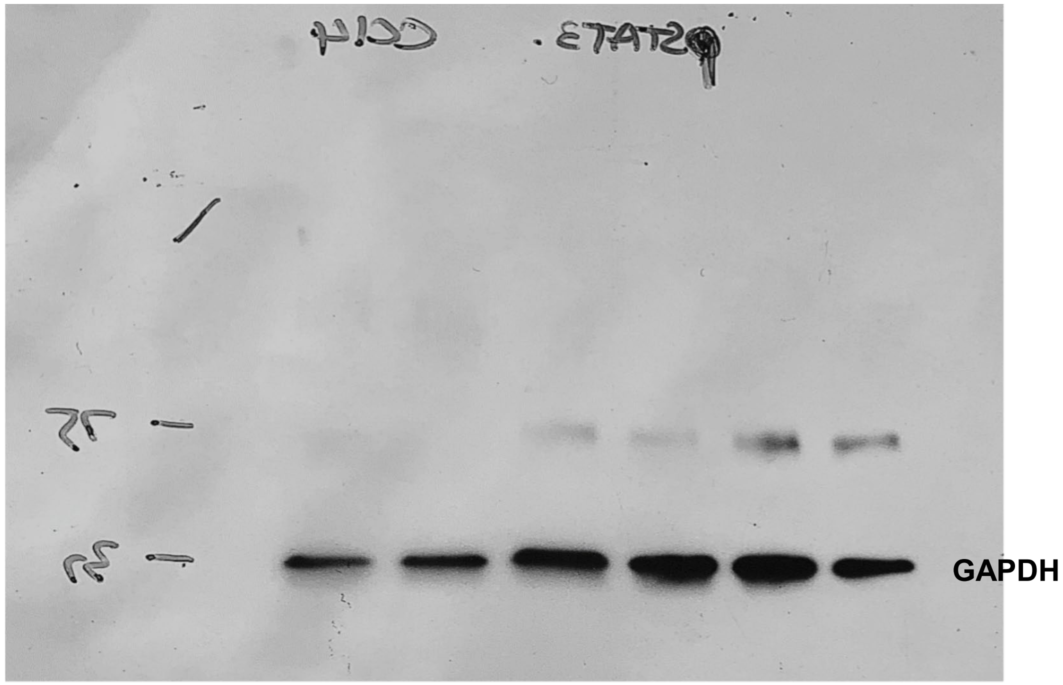

Fig 5B

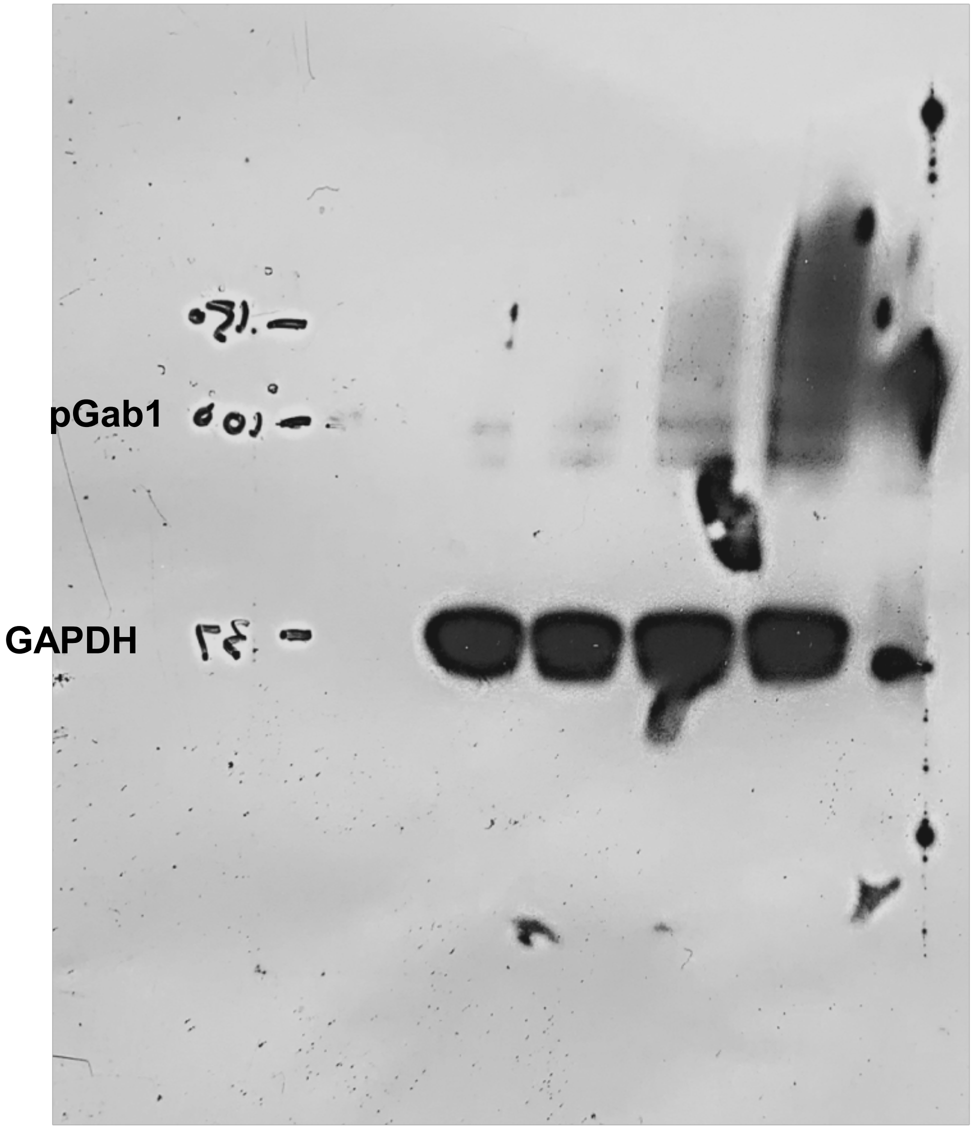

Fig 6B

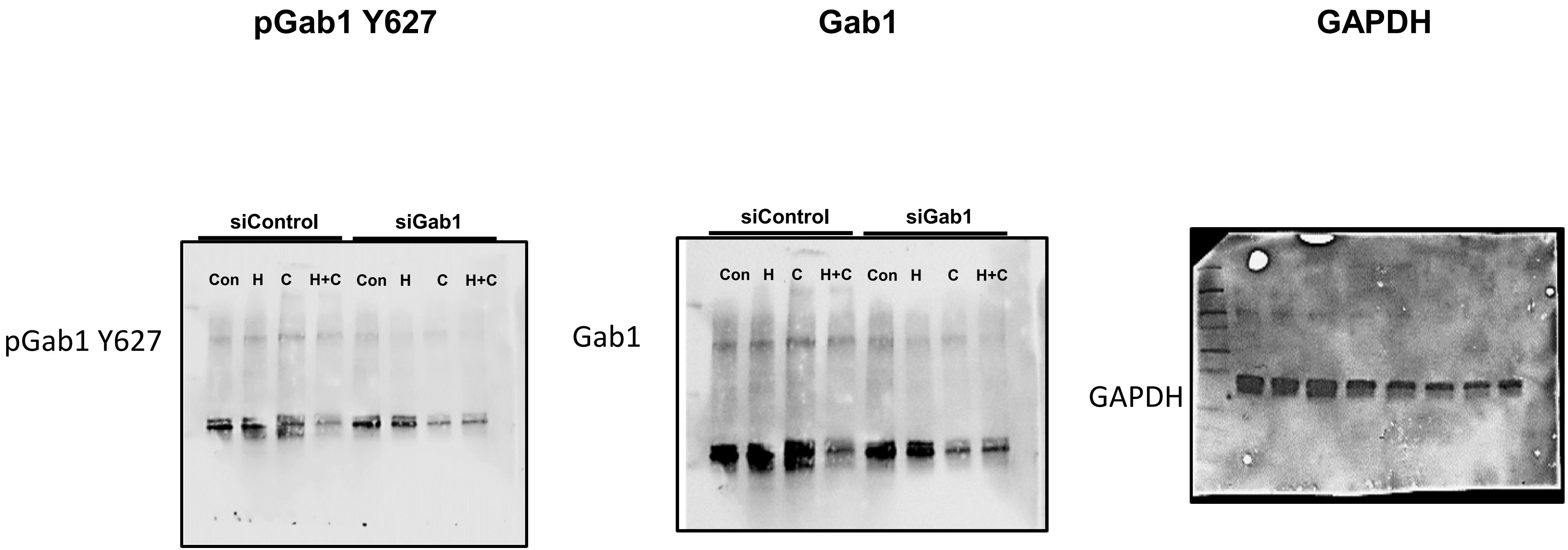
pGab1 Y627
Gab1
GAPDH
siControl
siGab1
Con H C H+C Con H C H+C
pGab1 Y627
siControl
siGab1
Con H C H+C Con H C H+C
Gab1
GAPDH

Fig 6C

PSTAT3 (79/86kD)

pERK1/2 (42/44kD)

GAPDH (37kD)

Pro-Caspase-3

Cleaved-Caspase-3

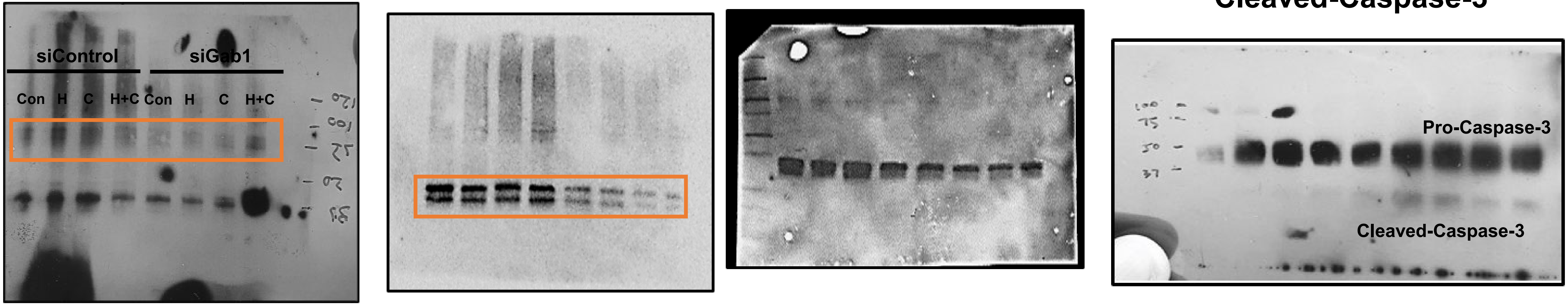

Supplement: S1 Raw images — (PDF) [file pone.0306345.s003.pdf]
