# Supplementary material for: Activated Gab1 drives hepatocyte proliferation and anti-apoptosis in liver fibrosis via potential involvement of the HGF/c-Met signaling axis
Source: PLoS One. 2024 Jun 27;19(6):e0306345. doi: 10.1371/journal.pone.0306345 (PMC11210754; doi:10.1371/journal.pone.0306345)
Supplement: Attachment — Submitted filename: Response to Reviewers.docx [file pone.0306345.s004.docx]

We appreciate for reviewers’ efforts and consideration of the manuscript for publication. Our responses to reviewers’ comments are described below.

**Response to Reviewer 1’ comments**

The authors have presented a well written and rigorously conducted experiment. Figures and tables have been provided to demonstrate the results and statistical analyses conducted. The conclusion obtained from the study provides important insight into the role of Gab-1 in the therapeutics of chronic liver disease.

Response: We appreciate for the reviewer’s positive comments on the manuscript.

**Response to Reviewer 2’ comments**

**Comment 1**

Materials and Methods – It was not mentioned how picrosirius-stained regions were measured. Equipment? Methodology?
.

Response: We added the contents related to picrosirus red staining in Methods section (line 194-199)

**Comment 2**

Results – Lines 292-293 – Apparently, it cannot be assumed that mRNA expression of ERK has increased. No statistical significance was indicated in Figure 2. Similarly, increase in cleaved caspase-3 cannot be attributed because p = 0.31 is not accepted to mean statistical significance (Line 297).

Response: We added the sentence related to “There’s no statistical difference in mRNA expression of ERK” at line 296.

Line 327 and Figure 3A – Apparently, a significant increase of Gab1 mRNA occurred until D1.

Line 330 and Figure 3D – The same consideration applies to ERK.

Response: “D2” was revised as “D1” in line 331.

Fig. 4 – E does not refer to mRNA expression. Figure elements should be described sequentially in the text. Description of D should precede E or restructure of the position of the figure elements should be undertaken.

Response: The order of the Fig 4D, E, and F has been changed for sequential explanation (Fig4 and line 369-372).

Fig. 5 – There is something wrong with (C); the column named Merge shows only DAPI staining. Legend: There is no ***P< 0.001 in this figure

Response: We revised “Merge” as “DAPI”, and deleted “***P< 0.001” in the Fig.5 legend. (line 431-432)

Fig. 6 – (E) Insert “Liver damage” over the line where “Early phase” and “Late phase” appear written. Legend: *P< 0.05 and ***P< 0.001 do not refer to this figure.

Response: We inserted “Liver damage” into the Fig6E. and we also deleted “*P< 0.05” and “ ***P< 0.001” in the Fig6 Legend. (line 447)

**Comment 3**

Minor:
. Standardize: min or minutes

Response: “minutes” was revised as “min” in line 99 and 155.

. Lines 128, 130, 133, 134 … 3 mm, 10 min, 30 min, 100 µm, … respectively

Response: All spacing correction has been completed. (line 128, 130, 133, 134)

. Line 137 – Insert “assay” after blue

Response: We added the “assay” after blue in line 137.

. Lines 146, 147, 243 – The first time the commercial source is mentioned, please add city and country

Response: We added the city and country the first time a commercial source was mentioned. (line 145-146)

. Line 154 – “mm2”

Response: We changed the “2” as superscript in line 155.

. Lines 160, 375,395,402 – typos

Response: We didn’t find mistake on spelling in line 160, 375, 395, 402.

. Line 171 – “described [17]”

Response: We added a space between “described” and “[18]” (ref 17 was changed as 18) in line 171.

. Line 178 – a verb is missing

Response: We revised the sentence including a verb like “There were 150 antibodies used in the Reverse-phase protein microarray data study (Table 1).” in line 177-178.

. Lines 246-247 – Here, (Sigma, Aldrich) is sufficient. City and country should be added to Sigma mention at line 147.

Response: We revised it as “Sigma-Aldrich” in line 250.

References – 1. Please abbreviate the name of the journal

Response: The name of the journal was corrected as abbreviation.
